# Online Education for Undergraduate Health Professional Education during the COVID-19 Pandemic: Attitudes, Barriers, and Ethical Issues

**DOI:** 10.21203/rs.3.rs-42336/v1

**Published:** 2020-07-16

**Authors:** Suhaib Muflih, Sawsan Abuhammad, Reema Karasneh, Sayer Al-Azzam, Karem H Alzoubi, Mohammad Muflih

**Affiliations:** Jordan University of Science and Technology; Jordan University of Science and Technology; Jordan University of Science and Technology; Jordan University of Science and Technology; Jordan University of Science and Technology; Jordan University of Science and Technology

**Keywords:** Health professionals, Undergraduate students, Online education

## Abstract

**Background:**

The online teaching demand has increased tremendously to promote the implementation of online teaching-leaning system to meet the need of students during the outbreaks of emerging infectious disease. This study aims to explore whether the pandemic of COVID-19, which requires universities to rapidly offer online learning, will affect attitudes about online education for undergraduate health sciences students. Also, it investigates the barriers for using online tools.

**Method:**

A cross-sectional survey using online social media was used to recruit eligible participants. The data for this study were focused on students’ experiences utilizing an online education method offered by the Jordanian government universities. This study is utilizing newly developed measuring tools that are expected to enable students to evaluate online teaching in terms of their own learning progress.

**Results:**

A total of 1,210 participants agreed to complete the online survey questionnaire. The mean score preparedness and attitude toward online education was average. The majority of students agreed that online courses helped assign reading and homework time better than on-campus approach (75.0%) and felt comfortable to actively communicate with my classmates and instructors online. Zoom and eLearning were the most common online platforms utilized by students. The geographic locations, lack of past experience on using online tools, and lack of past experience on using online tools were identified by students as the main barrier to online educations.

**Conclusions:**

Although the pandemic of COVID-19 appeared as uncommon catalyst for promoting eLearning, further research is needed to assess whether learners are ready and willing to make greater use of online education to obtain high quality teaching and learning opportunities, which could totally change educators’ and students’ attitudes and impression, and subsequently the general themes of online education.

## Introduction

As technology has been incorporated into the educational process, the online education demand has increased tremendously to promote the accessibility, feasibility, and implementation of online learning-leaning system to meet the need of students as well as faculty members [1]. Due to the wide availability of direct Internet connections, eLearning is frequently recognized as web-based learning [2, 3], moving on-campus learning to a distance education model, which can be implemented as a self-paced independent study style, asynchronous interactive gatherings, or real-time interactive settings [4]. During the current pandemic of COVID-19, most professors and students unexpectedly find themselves responsible for learning courses that have not been designed for online delivery before. Thus, universities’ professors and students started voluntarily experiencing and navigating into academic cyberspace, which allows them to interact with instructions in virtual settings. Although the pandemic of COVID-19 appeared as uncommon catalyst for promoting eLearning, it is still unclear whether students are ready and willing to make greater use of online education to obtain high quality learning and learning opportunities, which could totally change students’ attitudes and impression, and subsequently the general themes of online education.

Several studies suggested that online and blended educational approaches can be comparable to that traditional classroom models, nevertheless limited number of studies have focused on students’ and professors’ satisfaction with online education during pandemic crisis. Smart & Cappel (2006) revealed that time required to complete the online modules designed for undergraduate courses was the most common dissatisfaction factor for online education( Smart & Cappel, 2006). However, the overall satisfaction was positive in the online elective course. Studies showed that web-based courses have the potential to create learning environments that actively engage students with their material to build new knowledge [6, 7]. Additionally, eLearning can potentially provide many advantages in terms of flexibility and convenience to complete learning requirements, reduced overall cost, and provide an efficient way to communicate with students and capitalize on their feedback [8, 9] Also, it has been reported that massive online courses can potentially lower the cost of education for a large number of students [10]. More important, differences among students should be taken into account to enhance the online education process as indicated by Terras and Ramsay (2015) [11]. Further, online education can be used to facilitate growth through interaction with peers and active participation [11, 12] The additional demands on faculties and students to implement online education can take them out of their comfort zones and that necessitate mastering of technical skills through receiving training sessions to facilitate learning and achieve learning goals.

Surveying teachers and students at the present time to figure out the levels of educators’ and students’ preparedness and attitude towards online education and who will need devices and instructions could potentially speed up the adoption and implementation of online education to efficiently transform class-based learning into simulated settings in this compressed time frame. Further, the findings of this work could set back or magnify students’ attitudes about the quality of web-based learning. Moreover, sharing students’ feedback about the learning experiences during the time of emergency could better emphasize the use of more technologies, document technology’s potential for improving learning, and enhance users’ attitudes toward technology integration into the instructional process. As it was important to inspire faculties and students to follow remote learning practices and share their learning experiences, students and faculty will benefit from the results of this study that aim to raise important considerations about using online education to best meet the learning needs of students.

The current proposed research expected to achieve the following objectives:

1. To evaluate the socio-demographic characteristics influencing students’ willingness and preparedness to succeed in online courses
2. To measure the level of students’ preparedness and attitude towards online learning
3. To evaluate factors those, impede the process of online learning.
4. To determine the predictors of attitudes towards online learning

## Method

A cross-sectional survey using online social media was used to recruit eligible participants. The data for this study are based on student’s experiences utilizing online education methods offered by the Information Technology Centers of Jordanian government schools Eligible participants were all students at Jordanian government universities. From April 1^st^ through May 1^st^, 2020 a campaign approach using a combination of online social media and Web-based survey software were implemented to advertise, recruit, survey participants, and collect data for this survey study. The authors used four methods to recruit participants on paid-advertised Facebook, personal messages, and postings in medical-focused forums. Before participants can agree to willingly participate in the study, participants need to click the attached link that directed them to the online survey.

A consent form attached online with the survey questionnaire needs to be signed voluntarily by the participants before completing the survey. Students agree to sign an informed consent; he/she complete a survey questionnaire online. It took them about 8 minutes to complete the survey. Participating students were notified that their participation may contribute to an increase in the understanding of the use of online education methods. Although no personal identifiable information was collected from participants, they were assured that the survey data are protected due to the strict privacy and confidentiality procedures of the study.

### Instruments

The survey consists of Three parts, namely, sociodemographic characteristics, preparedness and attitudes toward the online learning, and potential barriers. This part has two parts attitudes towards online education (19 items) and preparedness of online education platforms (7 items). The response of each item ranged from 1 to 3. Agree (1), neutral (2) and disagree (3). The second part is barriers to use online education tools (9 items). The response of each item ranged from 1 to 3. Agree (1), neutral (2) and disagree (3). The survey items were developed based on extensive literature review and previously validated scales[13–17]; besides, some items were developed based on qualitative information collected during pilot testing stage. A panel of eight experts in educational technology and socio-behavioral sciences was invited to evaluate the items to assess the face and content validity of the newly developed scales, ensuring that the final survey item pool reflects the desired constructs of interest. The instrument was verified to have internal consistency based on the results of Cronbach alpha coefficient. The Cronbach alpha was for subscales as following online education (0.73), preparedness of online education platforms (0.68), and barriers to use online education tools (0.78).

### Ethical Consideration

The Jordan University of Science and Technology (JUST) IRB approved this research. Eligible participants were given detailed information about the goals of the study as well as the risks and benefits that may result from participation. The participants were instructed to read the informed consent form so that they can decide whether or not to participate in the current anonymous survey research.

### Statistical Analysis

Statistical packages IBM® SPSS version 24.0 was utilized. Descriptive statistics were used in describing the demographics of the research subject and the total attribute scores. Means, ranges, medians and standard deviations were used as continuous variables, while percentages and frequencies were used for grouped measures. All the assumptions of multiple regressions were checked.

## Results

### Demographic statistics

A total of 1210 students agreed to participate in the study. The vast majority of participants were female students (80.5%). Almost two-thirds of participants were 21 years old or younger. Almost half (59.3%) of students were pursuing a bachelor’s degree in pharmacy or Doctor of Pharmacy. The socio-demographic characteristics of participants are shown in Table 1.

### Online Education Tool

Many tools were used during online educationZoom, eLearning/School Portal, Moodle, Microsoft Teams, Email, Google Classroom, Online forum, WhatsApp, and Facebook. The most used instrument was eLearning (N = 1073, 88.7%) followed with Microsoft office team (N = 992, 82%). The highest rank and score for the effectiveness of the tool was given to YouTube (N = 639, 52.81 %) and eLearning (N = 473, 38%).

### Preparedness And Attitude Toward Online Learning

The mean score of preparedness and attitude toward online education was (M = 42.94, SD = 7.92). Many students agreed that “Online courses help me assign reading and homework time better than on-campus approach (N = 865, 71.5%), I feel comfortable to actively communicate with my classmates and instructors online (N = 808, 66.8%), and they agreed “I can ask my teacher questions and receive a quick response online” (N = 757,62.6%). Only a few students agreed that “I prefer face-to-face (in class) contact with my instructor for more efficient learning ( N = 171, 14.1%) and that “I have satisfactory computer skills for dealing with online course/assignments (N = 183, 15.1 %). Many students agreed that may preparation are required include basic computer literacy course (N = 340, 28.1 %), skills training in using computers and the internet ( N = 493, 40.7%) and training to help receive online course ( N = 491,40.6%). See Table (2)

### Barriers Toward Online Learning

Based on our study the main barriers of online education according to students’ responses (M = 13.1, SD = 4. (3 were “Living close to educational institutions” (N = 998, 82.5%), “Lack of technology experience” (N = 909, 75.1 %) and “Lack of past experience on using online tools” (N = 899, 74.3%). Few students agreed that major barriers of using technology “Lack of motivation” (N = 694, 57.4%) and “Inability to networking with expert in the field” (N = 759, 62.7%). See Table (3).

### Predictors Of Preparedness And Attitude Toward Online Learning

Multiple regressions test was conducted to determine the factors that had an impact on the level of student preparedness and attitude toward online learning. The model was significant (F = 89.97, p = 0.01). The factors that most affected the student preparedness and attitude toward online education were years in major (t = 3.03, p = .01), prior experience in learning (t = 3.30 P = .01), well-prepared to join online education (t = 22.33, P = .01) and perceived barriers (t= −9.478, P = .01). As decrease number of barriers the preparedness and attitude toward online education were increased. The other factors were not found to be preparedness and attitude toward online learning. See Table (4)

## Discussion

According to our knowledge, this is the first study in Jordan examining the preparedness and attitudes of students towards online education during the pandemic COVID-19. This research aims to review the socio-demographic attributes of students’ willingness to achieve in online education, gauging the degree of students’ attitudes regarding online education, identifying online education barriers, and evaluating factors that prevent online education.

Based on our study, students faced numerous technological challenges in online education. The findings were similar to other research conducted in universities in developing countries such as Saudi Arabia, for example, Alzahrani (2015) [18], and Tashkandi and Al-Jabri (2015) [19], who concluded that students in Saudi Arabic universities use educational technology in learning. Additionally, Alshwaier et al. (2012) [20], made a similar conclusion and that the students had satisfaction with various and consistent services from Google Educational Apps. The research question finding has consistency with the study of Lis and Paula (2015) [21], at Czestochowa Technical University, who indicated that 89% of the students used educational technology effectively in learning.

### Readiness and attitudes towards online education

Based on our study, our students had low readiness and a negative online education attitude. This was not consistent with the study Alhazzani (2014) [22] conducted such a study at King Saud University, to assess the benefits and disadvantages of Saudi online education from the faculty and students viewpoint, and to evaluate the utilization level of online education instruments by surveying 200 departmental members and their students. According to the findings of this study, 56.7% of the respondents had familiarity with the online education concept, 6.7% were noncommittal on the idea, while 3.3% had a strong familiarity with the online education concept. According to the findings, a large participant proportion (96.7%) admitted that the online education idea is undoubtedly a big leap towards the growth in the system of higher education in developing nations. Besides, many respondents stated various online education benefits, for instance, offering convenient application access.

Similarly, a study conducted in three Thailand public universities that investigated the preparedness for online education in a student sample. According to the findings, the online education and preparedness level among participants was above average. Teo (2009) [23] concluded that the favorable perception of users on their capacity to use technology significantly influenced their objective to use technology positively. Besides, the perception of an individual’s ability to apply technology also confidently and substantially indirectly affects the tendency of technology use through recognized benefits and recognized simplicity of application. This impacts progressing learning and permanent Thailand’s educational policies.

Nonetheless, after a short training of students in CCT use during learning, he found that the student’s desire to embrace this technology to improve their learning had increased. Nevertheless, some barriers prevented the acceptance of the new technology, for example, utilization requirements and inadequate resources. Alanazy (2011 ) [24] examined the attitudes and beliefs of Saudi students towards using coeducational online partnership learning in developing nations. The study findings showed that the opinions of Saudi students towards the application of coeducational online collaborative learning are generally favorable. Saudi students in the United States who have experienced a coeducational online partnership education setting believe that it is possible and right to use this setting in developing nations. Furthermore, they think that this setting will succeed if used in developing countries.

Nevertheless, since online education worldwide seems to be an appropriate answer to many institutions of higher education, and since few studies have been carried out in developing nations to learn the adoption and use of the new technology to improve learning, this study will delve deeper to investigate the attitudes of students towards the utilization of online education technology to promote collaborative learning in the higher learning institutions in Jordan.

The necessary preparation of students before the delivery of online courses can promote the learning of a student. Our study found that many students support that they required Skills training in using computers and the Internet and training to help receive your online course. Nevertheless, according to Maclaren & Shukla (1999) and Zsohar & Smith (2008) [25,26], preparation courses must have clear guidelines in their design to guarantee adequate preparations for both the students and their instructors before their commencement. A study conducted in first year found that students lacked an initial understanding of online education. The lack of knowledge of technology and computer, therefore, meant that it is essential for colleges to develop online courses using a system that is clear and easy to navigate, which focuses on the learning preferences of students to include students who are beginning to learn computers. This study found that a higher percentage of undergraduate health students lacked prior understanding of online education. Offering adequate training on primary computer skills and online education will guarantee that all undergraduate health students succeed in any of the many courses the students’ register.

### Barriers of online learning

Based on our study, the significant barriers to online education are closer residence to learning institutions, and inadequate experience in online tools use. According to a survey carried out in four universities in Thailand, students experienced significant challenges in e-learning. According to Siritong et al (2006) [27] all the interviewed instructors possessed little or no experience in online education methods. Therefore, they did not have confidence in e-learning implementation. From the perspective of students, they indicated that poor availability of points of access, slow communication networks, and lack of application software to be their barriers to engage in e-learning. Saekow and Samson (2011 ) [28] concurred with these results since they found that instructors and their students in Thailand had failed to recognize the benefits and lacked interest in enforcing e-learning, while students lamented the inadequate educational and technology resources.

According to Tashkandi and Al-Jabri (2015), additional barriers included limited internet bandwidth, means of communication, and is expensive. A respondent in this study stated that “there is no problem with online learning, but rather with the means of communication.” Concerns of data are an additional issue that influences the acceptance of online education. Data concerns mean concerns regarding the exposure of confidential information, illegal access of learner and research information, and losing other delicate information [29]. According to Tashkandi and Al-Jabri (2015), “data concern is generally considered an barrier in the acceptance of online learning” (p.1530)., they contend that exposing the system to the internet has some concerns, as a result of sharing physical infrastructure with various clients, and data management by the service provider because public online education depends on sharing. Different server data storage concern was among the drawbacks. Moreover, many respondents stated other drawbacks, including publishing policy concerns and property rights, stability, and essential data security. Further disadvantages included management, facilities, and financial challenges. Alzahrani (2015) conducted a study at Albaha University, a Saudi university, to examine the truth of technology instruments in higher education learning. According to his findings, 50% of his study sample were either unaware of online education or had poor experiences.

### Determinants of online education attitudes

This study found that the number of years in major of a student is a predicator that can determine their e-learning attitudes. The decisive point of view can be credited to these two courses’ characteristics since they frequently expose students to online education units.

The findings of this study did not find any statistically significant variation between the perspectives of male and female students towards online learning, which had inconsistency in past research [30,31] Both male and female students had a positive view of the advantages of online education in settings of online learning. The results of Kim et al (2013) [31] revealed a preference difference between male and female students since female students exhibited stronger desires regarding expression, imagination, and development compared to male students. A probable cause of result variation may because of differences in the numbers of male and female students in this research compared to study by Kim (2013) [31]. While the male proportion in my study sample accounted for just 19.3% of all students, the study sample of 66.7% males in Kim (2013) [31].

### Ethical issue in online education

The participating students highlighted a moral aspect in using online education, namely, the ability to access the Internet, which is very vital for the continuity and success of the eLearning process. Only 16.5% were able to conveniently access the Internet for their studies. Besides, healthcare professional students were not receptive to using the Internet for successful completion of online courses as the majority of participants reported lack of experience (75.1 %) and motivation (57.4%) regarding online learning, which might negatively affect the learning outcomes. Students who had skills in using the Internet and different online tools were concerned about the lack of instructors’ experiences to use online tools, which raised ethical issues particular to online education and interactions between students and instructors.

Moreover, this study did not provide clear evidence to support the role of the Internet and eLearning in social inclusion, as small percentages of participants felt comfortable taking classes sharing their thoughts online. Thus, further enhancements and assessments are required to ensure that the advantages of online education will also include learners who are socially or economically disadvantaged. Finally, the majority of courses shifted to online education during the COVID-19 pandemic were not designed to enhance students’ cultural competence, and that might increase the potential for miscommunication among students and instructors in online discussions. The findings of this research suggest that careful consideration of the issues ethical issues related to matters of equity and diversity is highly required.

### Implications for the future

The study findings have implications for decision-makers, instructors, leaders, and administrators of Universities in Jordan on education improvements. Cloud computing can enhance collaborative learning and learning and a useful solution for the education system.

Cloud computing is economically suitable for higher learning institutions as it reduces expenses.

## Conclusion

In summary, the geographic locations, lack of past experience on using online tools, and Lack of past experience on using online tools were identified by students as the main barrier to online educations. Although the pandemic of COVID-19 appeared as uncommon catalyst for promoting eLearning, further research is needed to assess whether learners are ready and willing to make greater use of online education to obtain high quality teaching and learning opportunities, which could totally change educators’ and students’ attitudes and impression, and subsequently the general themes of online education.

## Supplementary Material

Supplement

## Figures and Tables

**Table 1:** Frequency Distribution of Sociodemographic Characteristics of Participants (n = 1210)

Variable	Frequency (%)
Gender	
Male	236 (19.5)
Female	974 (80.5)
Age group	
18–19	325 (26.9)
20–21	504 (41.7)
22–23	324 (26.8)
Above 23	57 (4.7)
College Level	
Freshmen (first year)	267 (22.1)
Sophomore (second year)	229 (18.9)
Junior (third year)	306 (25.3)
Senior (fourth year)	150 (12.4)
Senior (fifth year)	211 (17.4)
Senior (sixth year)	47 (3.9)
The College Degree	
Medicine	492 (40.7)
Bachelor of Pharmacy	199 (16.4)
PharmD	519 (42.9)
Area of living	
Urban	783 (64.7)
Rural	427 (35.3)
Prior Experience with Online education	
Yes	456 (37.7)
No	754 (62.3)

**Table 2:** Description of attitude and preparedness of students toward online learning

	Agree		Neutral		Disagree	
	Count	Row N %	Count	Row N %	Count	Row N %
You are well-prepared to join online learning	241	19.9%	321	26.5%	648	53.6%
Before the emerging of COVID-19, the university used to support online education	379	31.3%	353	29.2%	478	39.5%
After the emerging of COVID-19, the university started supporting online education	755	62.4%	292	24.1%	163	13.5%
Online education enables students to continue their education similar to the traditional approach	206	17.0%	322	26.6%	682	56.4%
With the existence of online education, pandemic does not disrupt my future plans	232	19.2%	412	34.0%	566	46.8%
My university delivers a high-quality online education experience	210	17.4%	179	14.8%	821	67.9%
I would prefer to have online education to become the new normal	383	31.7%	267	22.1%	560	46.3%
I believe that faculty members will overcome the challenges of online learning	234	19.3%	255	21.1%	721	59.6%
I am able to easily access the Internet for my studies	200	16.5%	218	18.0%	792	65.5%
I feel comfortable taking online courses	272	22.5%	224	18.5%	714	59.0%
I feel comfortable to actively communicate with my classmates and instructors online	808	66.8%	204	16.9%	198	16.4%
I feel that my background and experience will facilitate my involvement in online studies	419	34.6%	360	29.8%	431	35.6%
I feel that taking courses online will help me to remember/master them better.	211	17.4%	413	34.1%	586	48.4%
Online courses help me assign reading and homework time better than on-campus approach	865	71.5%	199	16.4%	146	12.1%
I am able to complete assignments on time	258	21.3%	352	29.1%	600	49.6%
I prefer in-class approach as it provides a lot of interaction with my instructors and students	242	20.0%	391	32.3%	577	47.7%
I have satisfactory computer skills for dealing with online course/assignments.	183	15.1%	216	17.9%	811	67.0%
I feel more comfortable sharing my thoughts in an online education environment than in-class	313	25.9%	469	38.8%	428	35.4%
I can ask my teacher questions and receive a quick response online	757	62.6%	220	18.2%	233	19.3%
I prefer face-to-face (in class) contact with my instructor for more efficient learning	171	14.1%	287	23.7%	752	62.1%

**Table 3:** Barriers of online education faced by students

	Agree		Neutral		Disagree	
	Count	Row N %	Count	Row N %	Count	Row N %
Limited technology experience	909	75.1%	185	15.3%	116	9.6%
Lack of past experience on using online tools	899	74.3%	208	17.2%	103	8.5%
Lack of motivation	694	57.4%	301	24.9%	215	17.8%
Too challenging eLearning materials	738	61.0%	268	22.1%	204	16.9%
Lack of instructions	822	67.9%	222	18.3%	166	13.7%
Avoiding commonly used online tools such YouTube, Facebook by instructors	863	71.3%	220	18.2%	127	10.5%
Living close to educational institutions	998	82.5%	148	12.2%	64	5.3%
Inability to networking with expert in the field	759	62.7%	245	20.2%	206	17.0%
Too challenging eLearning tools	766	63.3%	229	18.9%	215	17.8%

**Table 4:** Predictors of attitudes toward online learning

Model		Unstandardized Coefficients		Standardized Coefficients	t	Sig.
		B	Std. Error	Beta		

1	(Constant)	26.666	2.899		9.198	.000
	
	Gender	.329	.451	.016	.728	.467
	
	Age	.342	.134	.083	2.548	.111
	
	Major	−.031	.039	−.019	−.797	.425
	
	Years of the Program	−.557	.184	−.102	−3.019	.003
	
	Type of living area	.524	.362	.031	1.449	.148
	
	Do you have a prior experience with online education)	1.196	.362	.073	3.307	.001
	
	The number of online courses for the current semester	−.001	.002	−.011	−.531	.595
	
	The number of hours you spend online for education	2.186E-012	.000	.008	.390	.697
	
	Indicate number of hours online general	.006	.009	.014	.658	.511
	
	You are well-prepared to join online learning	5.569	.249	.549	22.333	.000
	
	Barriers	−.453	.048	−.231	−9.478	.000

a. Dependent Variable: SUMAA

## References

[1] Appana S. A review of benefits and limitations of online learning in the context of the student, the instructor, and the tenured faculty. Int J E-Learning. 2008;7(1):5–22.

[2] Wasim J, Sharma SK, Khan IA, Jamshed S. Web- based learning innovations. Int J Comput Sci Inf Technol. 2014;5(1):5859–64.

[3] Lee BC, Yoon JO, Lee I. Learners’ acceptance of e-learning in South Korea: Theories and results. Comput Educ [Internet]. 2009;53(4):1320–9. Available from: 10.1016/j.compedu.2009.06.014

[4] Cook DA, Garside S, Levinson AJ, Dupras DM, Montori VM. What do we mean by web-based learning? A systematic review of the variability of interventions. Med Educ. 2010;44(8):765–74. 20633216 10.1111/j.1365-2923.2010.03723.x

[5] Smart K L., Cappel J J.. Students’ Perceptions of Online Learning: A Comparative Study. J Inf Technol Educ Res. 2006;5:201–19.

[6] Chen PSD, Lambert AD, Guidry KR. Engaging online learners: The impact of Web-based learning technology on college student engagement. Comput Educ [Internet]. 2010;54(4):1222–32. Available from: 10.1016/j.compedu.2009.11.008

[7] Ituma A. An evaluation of students’ perceptions and engagement with e-learning components in a campus based university. Act Learn High Educ. 2011;12(1 ):57–68.

[8] Hjeltnes TA, Hansson B. Cost Effectiveness and Cost Efficiency in E-learning [Internet]. Quality, Interoperability and Standards in e-learning. 2004 33 p. Available from: http://www2.tisip.no/quis/public_files/wp7-cost-effectiveness-efficiency.pdf

[9] Childs S, Blenkinsopp E, Hall A, Walton G. Effective e-learning for health professionals and students–barriers and their solutions. A systematic review of the literature-findings from the HeXL project. Health Info Libr J. 2005;22 Suppl 2:20–32. 10.1111/j.1470-3327.2005.00614.x 16279973

[10] Fain P As California goes? Insider Higher Education. 2013; Available from: http://www.insiderhighered.com/news/2013/01/16/california-looks-moocs-online-push.

[11] Terras MM, Ramsay J. Massive open online courses (MOOCs ): Insights and challenges from a psychological perspective. Br J Educ Technol. 2015;46(3):472–87.

[12] Furnes M, Kvaal KS, Høye S. Communication in mental health nursing - Bachelor Students’ appraisal of a blended learning training programme - An exploratory study. BMC Nurs. 2018;17(1):1–10. 29785174 10.1186/s12912-018-0288-9 PMC5952371

[13] Rajab M H, Gazal A M, Alkattan K (7 02, 2020) Challenges to Online Medical Education During the COVID-19 Pandemic. Cureus 12(7): e8966. doi:10.7759/cureus.8966. PMC7398724 32766008

[14] Keller C, Cernerud L. Students’ Perceptions of E-learning in University Education. J Educ Media. 2002;27(1–2):55–67.

[15] Link TM, Marz R. Computer literacy and attitudes towards e-learning among first year medical students. BMC Med Educ. 2006;6:1–8. 16784524 10.1186/1472-6920-6-34 PMC1534040

[16] Salter SM, Karia A, Sanfilippo FM, Clifford RM. Effectiveness of E-learning in pharmacy education. Am J Pharm Educ. 2014;78(4):16–22. 24850945 10.5688/ajpe78483 PMC4028592

[17] Panda S, Mishra S. E-Learning in a Mega Open University: Faculty attitude, barriers and motivators. EMI Educ Media Int. 2007;44(4):323–38.

[18] Al-Zahrani A. Toward Digital Citizenship: Examining Factors Affecting Participation and Involvement in the Internet Society among Higher Education Students. Int Educ Stud. 2015;8(12):203.

[19] Tashkandi AA, Al-Jabri I. Cloud Computing Adoption by Higher Education Institutions in Saudi Arabia: Analysis Based on TOE. 2015 Int Conf Cloud Comput ICCC 2015. 2015;

[20] Alshwaier A. A New Trend for E-Learning in KSA Using Educational Clouds. Adv Comput An Int J. 2012;3(1 ):81–97.

[21] Lis T, Paula B. The Use of Cloud Computing by Students from Technical University - The Current State and Perspectives. Procedia Comput Sci [Internet]. 2015;65(Iccmit):1075–84. Available from: 10.1016/j.procs.2015.09.050

[22] Alhazzani N. Students Interaction with E-Learning Environment (Blackboard) at King Saud University. Viteli J, Leikomaa M (Eds), Proc EdMedia 2014--World Conf Educ Media Technol (pp 417–431) Tampere, Finla 2014;

[23] Teo T. Evaluating the intention to use technology among trainee teachers using the Technology Acceptance Model: A structural equation modeling approach. 2009;5:106–18.

[24] Alanazy S. Saudi Students Attitudes Beliefs And Preferences Toward Coeduc. 2011;

[25] Maclaren P and Shukla H. DIVERSITY IN COURSE DELIVERY: PHARMACOLOGY 6 OFFERED ON CAMPUS AND IN A VIRTUAL CLASSROOM. Auckl Inst Technol New Zeal. 1999;

[26] ZSOHAR HELEN; SMITH JACKIE A. TRANSITION from the Classroom to the Web: Successful Strategies for Teaching Online, Nursing Education Perspectives: Jan-Feb 2008 - Volume 29 - Issue 1 - p 23–28. 18330418

[27] Siritongthaworn S, Krairit D, Dimmitt NJ, Paul H. The study of e-learning technology implementation: A preliminary investigation of universities in Thailand. Educ Inf Technol. 2006;11(2):137–60.

[28] Saekow Apitep and Samson Dolly. E-learning Readiness of Thailand’s UniversitiesComparing to the USA’s Cases. Int J e-Education, e-Business, e-Management e-Learning. 2011;1 (2).

[29] Oliveira T, Thomas M, Espadanal M. Assessing the determinants of cloud computing adoption: An analysis of the manufacturing and services sectors. Inf Manag [Internet]. 2014;51(5):497–510. Available from: 10.1016/j.im.2014.03.006

[30] Hamari J, Koivisto J, Sarsa H. Does gamification work? - A literature review of empirical studies on gamification. Proc Annu Hawaii Int Conf Syst Sci. 2014;3025–34.

[31] Kim D, Rueckert D, Kim D, Seo D. STUDENTS’ PERCEPTIONS AND EXPERIENCES OF MOBILE LEARNING Daesang. Lang Learn. 2013;17(3):52–73.

