# Supplementary material for: Online Education for Undergraduate Health Professional Education during the COVID-19 Pandemic: Attitudes, Barriers, and Ethical Issues
Source: Res Sq. 2020 Jul 16:rs.3.rs-42336. Preprint. [Version 1] doi: 10.21203/rs.3.rs-42336/v1 (PMC7373140; doi:10.21203/rs.3.rs-42336/v1)
Supplement: Supplement [file StudentSurveyElearning.docx]

**Online Learning**

**Student Questionnaire**

| Gender | Male | Female | |
| --- | --- | --- | --- |
| Age |  | | |
| Major | - Bachelor of Pharmacy - Pharm.D. - Medicine | | |
| Years of Program | - 1^st (^freshmen) - 2^nd^ - 3^rd^ - 4^th^ - 5^th^ - 6^th^ | | |
| Type of living area | - Urban - Rural | | |
| Do you have a prior experience with online education (e.g., attending webinars, receiving/giving online courses, online workshops, etc) | - Yes - No | | |
| Number of Online Courses for the current semester |  | | |
| Indicate number of hours you spend online (e.g., computer, smartphones, tabs) per week for educational purposes |  | | |
| Indicate number of hours you generally spend per week online for non- educational purposes |  | | |
| **Preparedness of students toward online learning** | | | |
|  | **Disagree** | **Neutral** | **Agree** |
| Are you well-prepared to join online learning |  |  |  |
| Before the emerging of COVID-19, the campus used to support online education |  |  |  |
| After the emerging of COVID-19, the campus start supporting online education |  |  |  |
| My school delivers a high-quality online learning experience |  |  |  |
| I believe that faculty members will overcome the challenges of online learning |  |  |  |
| I am able to easily access the Internet for my studies |  |  |  |
| I have satisfactory computer skills for dealing with online course/assignments. |  |  |  |
| **Attitudes of students toward online learning** | | | |
|  | **Disagree** | **Neutral** | **Agree** |
| Online education enables students to continue their education similar to the traditional approach |  |  |  |
| With the existence of online education pandemic does not disrupt future plans |  |  |  |
| Would you prefer to have online learning to become the new normal |  |  |  |
| I feel comfortable taking online courses |  |  |  |
| I feel comfortable to actively communicate with my classmates and instructors online |  |  |  |
| I feel that my background and experience will facilitate my involvement in online studies. |  |  |  |
| I feel that taking courses online will help me to remember them better. |  |  |  |
| Online courses help me assign reading and homework time better than on-campus approach |  |  |  |
| I am able to complete assignments on time |  |  |  |
| I prefer in-class approach as it provides a lot of interaction with my instructors and students |  |  |  |
| I feel more comfortable sharing my thoughts in an online learning environment than in-class |  |  |  |
| I can ask my teacher questions and receive a quick response online |  |  |  |
| I prefer face-to-face contact with my instructor for more efficient learning |  |  |  |
| I feel more motivated to register in online courses |  |  |  |
| I feel more motivated to teach my courses online |  |  |  |
| I can easily work in a group during online activities. |  |  |  |
| I feel that learning in class is better than on the Internet. |  |  |  |
| I can better practice English during online classes and activities than in-class |  |  |  |
| I believe all my degree courses can be given online without difficulty |  |  |  |
| **The following are real barriers to the online learning process** | | | |
| - **Barriers** | **Disagree** | **Neutral** | **Agree** |
| - Limited technology experience - Lack of past experience on using online tools - Lack of motivation - Too challenging eLearning materials - Lack of instructions - Avoiding commonly used online tools such as YouTube, Facebook by instructors - Living close to educational institutions - Inability to networking with expert in the field - Too challenging eLearning tools - Others (please mention) |  |  |  |
| **Which of the following online tools you using** | **Yes** | **No** | **Not Sure** |
| Zoom |  |  |  |
| eLearning/School Portal |  |  |  |
| Email |  |  |  |
| Online forum |  |  |  |
| Facebook |  |  |  |
| YouTube |  |  |  |
| Others (please mention) |  |  |  |
